# Multiomics Analysis Reveals Insights into Potential Drivers of Pancreatic Islet Pathology in Type 2 Diabetes

**DOI:** 10.1021/acsomega.4c10637

**Published:** 2025-06-30

**Authors:** Madelyn C. Houser, Jonathan M. Anzules, Tyrome Sweet, Anja M. Billing, Young-Mi Go, Steven E. Bosinger, Johannes Graumann, Khaled Machaca, Andrew F. Stewart, Peter M. Thule, Dean P. Jones, Vicki S. Hertzberg, Susan A. Safley, Collin J. Weber

**Affiliations:** † Nell Hodgson Woodruff School of Nursing, 1371 Emory University, 1520 Clifton Road, Atlanta, Georgia 30322, United States; ‡ Independent Researcher, Merced, California 95340, United States; § Proteomics Core, 36579 Weill Cornell Medicine – Qatar, Education City, Doha 24144, Qatar; ∥ Department of Medicine, School of Medicine, Emory University, 201 Dowman Drive, Atlanta, Georgia 30322, United States; ⊥ Department of Pathology and Laboratory Medicine, School of Medicine, Emory University, 201 Dowman Drive, Atlanta, Georgia 30322, United States; # Diabetes, Obesity, and Metabolism Institute, 5925 Icahn School of Medicine at Mount Sinai, 1 Gustave L. Levy Place, Box 1152, New York, New York 10029, United States; ∇ Department of Surgery, School of Medicine, Emory University, 201 Dowman Drive, Atlanta, Georgia 30322, United States

## Abstract

Despite the high prevalence of type 2 diabetes (T2D), the mechanisms driving pathology in pancreatic islet β cells remain poorly understood. We utilized a multiomics approach to evaluate the transcriptional and biochemical makeup of islets from human organ donors with T2D and nondiabetic controls. Transcriptomic (*N* = 10), proteomic (*N* = 6), and untargeted high-resolution metabolomic (*N* = 10) data were analyzed individually and then integrated using sparse partial least-squares regression, and differential network analysis was performed. In individual data sets, 25 transcripts, 30 proteins, and 30 metabolites were differentially abundant between T2D and nondiabetic islets, representing some pathways not previously characterized in T2D islets including purine and pyrimidine, branched-chain amino acid, and histidine metabolism. Network analysis of integrated data sets highlighted disrupted relationships among features in T2D islets compared to those from nondiabetic individuals. Fatty and amino acid metabolism and immune activity were identified as prominent drivers of the distinctions in biochemical interactions in T2D networks. Our findings also suggested greater abundance and influence of industrial chemicals, including polychlorinated and polybrominated biphenyls, in T2D islets. This pilot study demonstrates that multiomics profiling can identify candidate molecules and mechanisms impacting islet cell activity in T2D, which could represent targets for therapeutic intervention.

## Graphical abstract

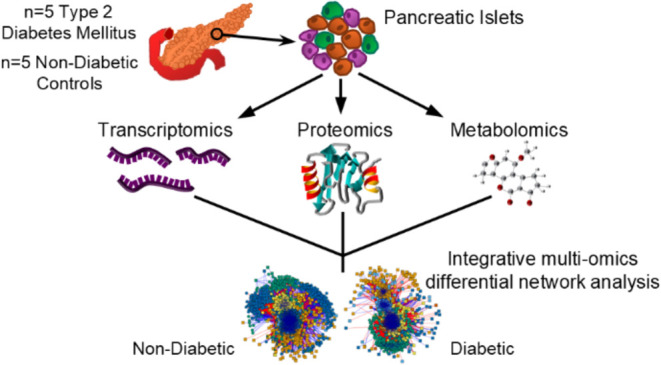

## Introduction

Type 2 diabetes mellitus (T2D) represents a major public health crisis of morbidity and mortality worldwide.^1^ In T2D, pancreatic islet β cells exhibit impaired insulin secretion in response to glucose. Peripheral resistance to insulin develops in muscle and adipose tissue, while central resistance develops in the liver.^2,3^ Chronic metabolic stress induces oxidative stress, endoplasmic reticulum stress, dedifferentiation to a precursor-like state, and apoptosis in β cells.^2,4–7^ β cell mass declines, although not typically to the extent observed in type 1 diabetes (T1D), and insulin secretion decreases while glucagon and somatostatin increase.^8,9^ The aggregate result is an absolute or relative insulin deficiency.

Despite extensive research on T2D, an understanding of T2D-related pathology in β cells remains incomplete. This is attributable to the heterogeneity of T2D manifestations, the complexity of T2D metabolic dysregulation, and the relative inaccessibility of pancreatic islets for study, which are primarily available from deceased donors. As such, there is a paucity of information on T2D islet β cells themselves, with a majority of research focusing on systemic alterations in patients.

Omics approaches, and particularly integration of multiple omics outputs, have the potential to better capture the breadth of physiologic abnormalities in conditions like T2D, as they provide extensive information on levels of molecules in a system in a relatively unbiased manner, enabling identification of novel markers and mechanisms of disease.^10^ In recent years, a number of studies have profiled islets from T2D donors by individual omics methods.^11–14^

Only a handful of studies have applied multiomics approaches to compare T2D and nondiabetic islets. These include studies integrating islet transcriptomics with peripheral plasma lipidomics,^15^ islet transcriptomics with single-cell chromatin accessibility,^16^ transcriptomics with proteomics of stimulated islets,^17^ and β cell transcriptomics with GWAS findings and islet function.^18^ These works primarily identified processes involved in insulin production as differentiators of T2D and nondiabetic islets. To our knowledge, only one other published study has integrated three omics data sets from pancreatic islets, utilizing a layered dimensionality-reducing approach on transcriptomic data, DNA methylation arrays, single-nucleotide polymorphism arrays, and demographic and phenotypic data.^19^ They found that an integrated model was more accurate in classifying T2D versus nondiabetic samples than individual data sets alone, with most of the key discriminatory features involved in insulin secretion.

To further explore the potential for multiomics to elucidate mechanisms involved in T2D islet pathology, we profiled a small set of T2D and nondiabetic islets using three omics methodologies individually (transcriptomics, proteomics, and metabolomics) and then utilized integrative network analysis to examine the three data sets in combination. By comparing networks from T2D and nondiabetic islets, we identified differences in gene, metabolite, and protein associations that are linked to processes beyond the well-known deficits in insulin secretion. These findings potentially offer insights into the biological processes driving T2D-related dysfunction in pancreatic islets.

## Experimental Methods

### Islets

Frozen islets from five T2D and five nondiabetic deceased donors were purchased from the Integrated Islet Distribution Program (IIDP). Islets from diabetic donors were isolated between September 2010 and April 2014, and islets from nondiabetic donors were isolated between July 2010 and March 2012 at the NIH Clinical Islet Transplantation Centers. Cells were cultured overnight and assessed for viability using fluorescein diacetate and propidium iodide and for percent purity using dithizone staining. The percentage of islets ranged from 70 to 90%, and islet viability ranged from 85 to 95%.

Islets were shipped to the lab in human islet medium (Connaught Medical Research Laboratories 1066, 25% Human Serum Albumin, Heparin Sodium 10 U/mL). Upon receipt, islets were decanted using the recommended IIDP protocol, centrifuged (170*g*, 3 min), washed, and resuspended in human islet media. Islets were aliquoted for each of the three assays, pelleted, flash-frozen, and stored at −80 °C until omics assays.

### Transcriptomics

RNA sequencing (RNA-Seq) analyses were conducted at the Emory National Primate Research Genomics Core. Cells were lysed by vortexing for 1 min in 350 μL of RLT/BME buffer, and total RNA was extracted using RNeasy mini kits (Qiagen) according to the manufacturer’s protocol for purification from animal cells with RNA cleanup. RNA yield and quality were evaluated by an Agilent 2100 Bioanalyzer G2939A. Polyadenylated transcripts were purified, reverse-transcribed using random hexamers, fragmented, and incorporated into barcoded complementary DNA libraries using the Illumina TruSeq Stranded mRNA Library prep protocol. Libraries were validated by microelectrophoresis, pooled, and sequenced on an Illumina HiSeq 1000, generating single-end reads.

Sequence quality was assessed by FastQC (version 0.11.9).^20^ The first seven nucleotides and Illumina adapter sequences were removed with Cutadapt (version 2.8).^21^ Salmon (version 1.10.0)^22^ was used to map subreads and quantify RNA expression levels, referencing the hg19 genome. A count matrix was produced using *tximport* (version 1.28.0).^23^ Of 20,643 genes identified, 15,295 were present in at least 80% of either T2D or nondiabetic donor islets. After excluding duplicate and unannotated genes, 14,832 were analyzed. The filtered count matrix was normalized, and differential gene expression analysis was performed using *DESeq2.* ^24^ Features with differential expression in T2D and nondiabetic islets were selected by the Wald test, *p* < 0.05. Those with adjusted *p* < 0.1 after controlling for false discovery rate (FDR)^25^ were noted.

Gene set enrichment analysis (GSEA) of differentially expressed genes was performed using *fgsea* ^26^ with random seed “06212022”. Genes were ranked by the product of the log fold change between groups and the negative log of the raw *p*-value and compared against two gene sets from the Human Molecular Signatures Database (MSigDB):^27^ Gene Ontology (GO) Biological Processes and Kyoto Encyclopedia of Genes and Genomes (KEGG) (accessed March 06, 2023). We restricted our analysis to pathways containing 6–500 genes. Leading up- and downregulated pathways were identified based on enrichment scores (ESs) and *p-*values.

### Proteomics

Frozen islets were transferred in two batches to the Research Division of Weill Cornell Medical College in Doha, Qatar, for proteomic evaluation. Due to complications with international shipping on dry ice, only one batch of islets, three from T2D and three from nondiabetic donors, could be assayed.

Cells were incubated for 30 min at room temperature in lysis buffer (2% sodium dodecyl sulfate [SDS], 10 mM 4-(2-hydroxyethyl)-1-piperazineethanesulfonic acid [HEPES] pH 8, protease inhibitors [Complete, Roche], phosphatase inhibitors [Sigma], benzonase [2 μL/mL, Sigma]), followed by sonication with five 3 s pulses at 10% amplitude. Lysed samples were frozen at −80 °C. Samples were thawed and underwent reduction (10 mM dithiothreitol, 1 h, 55 °C) and alkylation (15 mM iodoacetamide, 30 min, room temperature in the dark). Protein concentration was measured by the bicinchoninic acid assay, and sample integrity was verified by SDS-polyacrylamide gel electrophoresis. About 100 μg of protein per sample and 600 μg of a combined standard comprising a mixture of equal parts of all six samples were methanol/chloroform-precipitated and resuspended in 100 mM triethylammonium bicarbonate (TEAB) buffer (pH 8). Proteins were digested overnight at 37 °C with trypsin (1:50 enzyme:protein).

Samples were stable isotope dimethyl-labeled in TEAB buffer for 1 h at 22 °C using formaldehyde and cyanoborohydride. All samples were “heavy”-labeled (F–^13^C–D_2_, NaBD_3_CN); the combined standard was “light”-labeled (F–H_2_, NaBH_3_CN). Labeling was quenched with 1% ammonia solution followed by 1% formic acid solution. Labeled peptides were purified on Oligo R3 columns (Thermo Fisher) according to the manufacturer’s protocol, resuspended in deionized water, and fractionated by isoelectric focusing (20 kVh) overnight within a 3–10 pH range with 0.1% ampholytes and 0.3% glycerol using an Agilent OFFGEL Fractionator. Peptides were desalted by C18 StageTip solid phase extraction.^28^

Peptides were analyzed by liquid chromatography–tandem mass spectrometry (LC–MS/MS) using an EASY nLC-II coupled to a Q Exactive mass spectrometer, as previously described.^29^ MS raw files were trimmed with RecalOffline software (Thermo Fisher) to remove the first 30 min (excluding ampholytes) and then analyzed with MaxQuant (v1.4.1.2) software^30^ using default settings with match between runs and requantify options selected. Peptides in individual samples were measured as ratios to the combined standard. Searches were performed against the human UniProt database (download: May 15, 2014, 68,406 entries) with FDR = 0.01 for protein and peptide identification.

Of 7045 identified proteins, the 5323 detected in at least two-thirds of either T2D or nondiabetic islets were analyzed. Missing intensities below detection levels were replaced with half of the minimum value in the data set. The log_10_ transformation was applied. Proteins differentially abundant in T2D and nondiabetic islets were identified using *limma* ^31^ with *p* < 0.05. Those with adjusted *p* < 0.1 after FDR correction were noted. Patterns of protein abundance by sample were visualized by two-way hierarchical cluster analysis using Euclidean distance and Ward.D2 linkage in the *pheatmap* package.^32^

GSEA was performed using the same methods as described for RNA-Seq data. In the case of duplicated genes in the data set due to their association with more than one protein, the protein with the lowest limma *p*-value was retained.

### High-Resolution Metabolomics (HRM)

Untargeted high-performance HRM profiling of freshly thawed, frozen islets was performed by the Emory Clinical Biomarkers Laboratory using the methods described by Soltow et al.^33^ Acetonitrile (2:1, v/v) and 2.5 μL of internal standard mix (previously described^34^) were added to 50 μL of cell lysates. Proteins were pelleted, and supernatants were loaded onto a Shimadzu (Sil-20AC Prominence) autosampler. Ten microliters of each sample was resolved by an anion-exchange column (Hamilton PRP-X110S, 2.1 × 10 cm^2^) with a C18 precolumn (Higgins Analytical, Targa guard) using positive electrospray ionization mode and a formic acid gradient run at 0.35 mL/min. Data were collected by an LTQ-FT mass spectrometer (Thermo Fisher, San Diego, CA). Raw data files were converted to .cdf format using Xcalibur file converter software (Thermo Fisher, San Diego, CA), and apLCMS software^35^ was used for noise filtering, peak extraction, and ion intensity quantification.

Each sample was analyzed in triplicate, and the median was summarized. Of 23,322 extracted features, the 8842 found in at least 80% of either T2D or nondiabetic donor islets were analyzed. Missing intensities below detection limits were replaced with half of the minimum value in the data set. Differences in feature intensities between T2D and nondiabetic islets were identified using *MetaboAnalystR* (v3.1.0);^36^ log_10_ transformation and autoscaling (mean-centering each feature intensity and dividing by standard deviation) were performed, and differentially abundant features were selected by Welch’s *t* test, *p* < 0.05. Those with adjusted *p* < 0.1 after controlling for FDR were noted.

Pathway enrichment analysis was performed on metabolomic features selected by the *t* test at *p* < 0.05 with no FDR correction using the *mummichog* Python tool (v1.0.10),^37^ with 1000 permutations, “dpj” mode enabled, and the primary ion required. Pathways with adjusted *p* < 0.05 containing at least three differentially abundant metabolites were retained. Pathways in which at least 80% of differentially abundant metabolites were either more or less abundant in T2D islets were designated “upregulated” or “downregulated,” respectively. Pathways in which the differentially abundant metabolites did not meet these criteria were designated “mixed.”

*Mummichog* assigns tentative feature annotations based on a combination of mass:charge ratio (*m*/*z*) matching and feature clustering.^37^ *Mummichog* annotations were examined alongside those from the Toxin and Toxin Target Database (T3DB)^38,39^ to capture the potential impact of hazardous chemicals on islets. T3DB annotations were made by xMSAnnotator^40^ using a multistage clustering algorithm at five parts per million tolerance, as previously described.^41^

### Omics Integration and Differential Network Analysis

RNA-Seq, metabolomic, and proteomics data (filtered based on prevalence as described above) were integrated in a differential network analysis using *xMWAS*, which constructs networks and communities of features in them based on the strongest correlations among features.^42^ Non-normalized counts for RNA-Seq and proteomics data and missing value-imputed log_10_-transformed metabolomic feature intensity data were used. Separate networks were built for T2D and nondiabetic islets. Pairwise data integration and dimensionality reduction were performed using sparse partial least-squares (PLS) regression, with the number of features to include from each data set capped at 30% of the total in each data set, ranked according to relative standard deviation (random seed “08162023”). Optimal PLS component selection was calculated with a maximum of ten components. The threshold for pairwise correlation was 0.5 with a raw *p*-value of 0.05. Multilevel community detection was performed to identify clusters of related features.

Eigenvector centrality was used to evaluate and compare the level of connectedness of nodes in the T2D and nondiabetic islet networks, with higher eigenvector centrality for omics features that correlated in abundance with numerous other features that were, in turn, correlated in abundance with many other features. Features with high centrality are more likely to be directly related to, coregulated with, and/or influencing the abundance of numerous other features in the islets. Features with a difference in eigenvector centrality (delta centrality) >0.1 or <−0.1 between T2D and nondiabetic islet networks, as well as proteins linked with certain metabolites annotated as hazardous chemicals, were evaluated further utilizing *mummichog* and *fgsea* with the same parameters as described previously (except random seed “08162023” and GSEA ranks based on delta centrality or node weights reflecting closeness of linkages to putative hazardous chemicals) to identify biological process/pathways represented by these metabolites, transcripts, and proteins.

### Additional Analysis Tools

Unless otherwise specified, data were analyzed using the R statistical computing language (v4.0.2)^43^ and its integrated development environment, RStudio.^44^ In addition to packages specifically mentioned above, we utilized *tidyverse*,^45^ *RColorBrewer*,^46^ *matrixTests*,^47^ *ggpubr*,^48^ *qs*,^49^ and *data.table*.^50^

### Data Availability

The omics data and donor information are available from the Emory Dataverse repository (10.15139/S3/TFIX3Y).^51^

## Results

### Islet Cell Donor Characteristics

The characteristics of the islet cell donors are summarized in Table 1. Of the five nondiabetic donors, two were female, both under the age of 35. One diabetic donor was a female (age 57). Nondiabetic donors were, on average, younger than T2D donors (mean ages 38.6 and 50.4, respectively). Three nondiabetic donors were White, while two were “Black or African American.” Three diabetic donors were “Hispanic or Latino,” while the others are Asian or White. The majority of donors in both groups were categorized as overweight or obese per the body mass index (BMI). One donor in the nondiabetic group was underweight, and one donor in the diabetic group had a BMI categorized as healthy. Mechanisms of death were relatively balanced among the groups, with most deaths resulting from cardiovascular or cerebrovascular incidents and one death in each group attributable to head trauma.

**Table 1:** Islet Cell Donor Characteristics

	nondiabetic (*N* = 5)	diabetic (*N* = 5)	total (*N* = 10)
sex			
male	3 (60%)	4 (80%)	7 (70%)
female	2 (40%)	1 (20%)	3 (30%)
age (years)			
mean (SD)	38.6 (12.2)	50.4 (8.4)	44.5 (11.7)
min–max	22.0–54.0	42.0–61.0	22.0–61.0
race/ethnicity			
White	3 (60%)	1 (20%)	4 (40%)
Black or African American	2 (40%)	0 (0%)	2 (20%)
Hispanic or Latino	0 (0%)	3 (60%)	3 (30%)
Asian	0 (0%)	1 (20%)	1 (10%)
body mass index (BMI)			
mean (SD)	33.0 (11.7)	35.3 (10.1)	34.1 (10.4)
min–max	16.0–46.2	24.0–48.4	16.0–48.4
BMI classification			
underweight	1 (20%)	0 (0%)	1 (10%)
healthy weight	0 (0%)	1 (20%)	1 (10%)
overweight	1 (20%)	1 (20%)	2 (20%)
obese	3 (60%)	3 (60%)	6 (60%)
mechanism of death			
stroke	2 (40%)	4 (80%)	6 (60%)
cardiovascular—anoxia	2 (40%)	0 (0%)	2 (20%)
head trauma—blunt injury	1 (20%)	0 (0%)	1 (10%)
head trauma—gunshot	0 (0%)	1 (20%)	1 (10%)

Six of the ten islet samples were available for proteomic analysis from three male nondiabetic donors and two male and one female T2D donors. Ages in the subset ranged from 40 to 54 for the nondiabetic donors and 42 to 57 for T2D donors. All race/ethnicity categories used in this study were represented in the proteomics subset. Two-thirds of the donors in both groups in the proteomics subset had BMIs categorized as obese; the remaining nondiabetic donor was categorized as overweight, while the BMI of the remaining diabetic donor was categorized as healthy.

### Transcriptomics Highlighted Known Processes Related to T2D β Cell Dysfunction

Of 14,832 genes identified in transcriptomic analysis, 890 were differentially expressed in T2D vs nondiabetic islets at a raw *p*-value <0.05 (Figure 1A and Table S1) and 25 were differentially expressed after FDR adjustment (adjusted *p* < 0.1) (Figure 1B). Top differentially expressed genes by *p*-values were *AMY2A*, *KLF6*, *GARIN4*, *PPM1N*, *ATF3*, *CLPS*, *KLF10*, *MYLIP*, and *AMY2B* (Table S1). Differential expression of *GNMT*, *AMY2A/B*, *PLIN4*, *CLPS*, and *CELA3A* was heavily influenced by their expression in one patient (Figure 1B). High expression of several marker genes in this sample suggests that this islet isolation captured acinar pancreatic cells and β cells.^52^ Excluding this sample from the transcriptomic analysis resulted in the elimination of most of these acinar-cell-related genes from the list of differentially expressed features with the exception of *AMY2A*, which remained higher in the T2D samples. *KLF6*, *GARIN4*, *PPM1N*, and *ATF3* also remained significantly higher in T2D samples, and increased expression of the extracellular matrix gene *FBN2* was also identified (Table S1).

**Figure 1:**
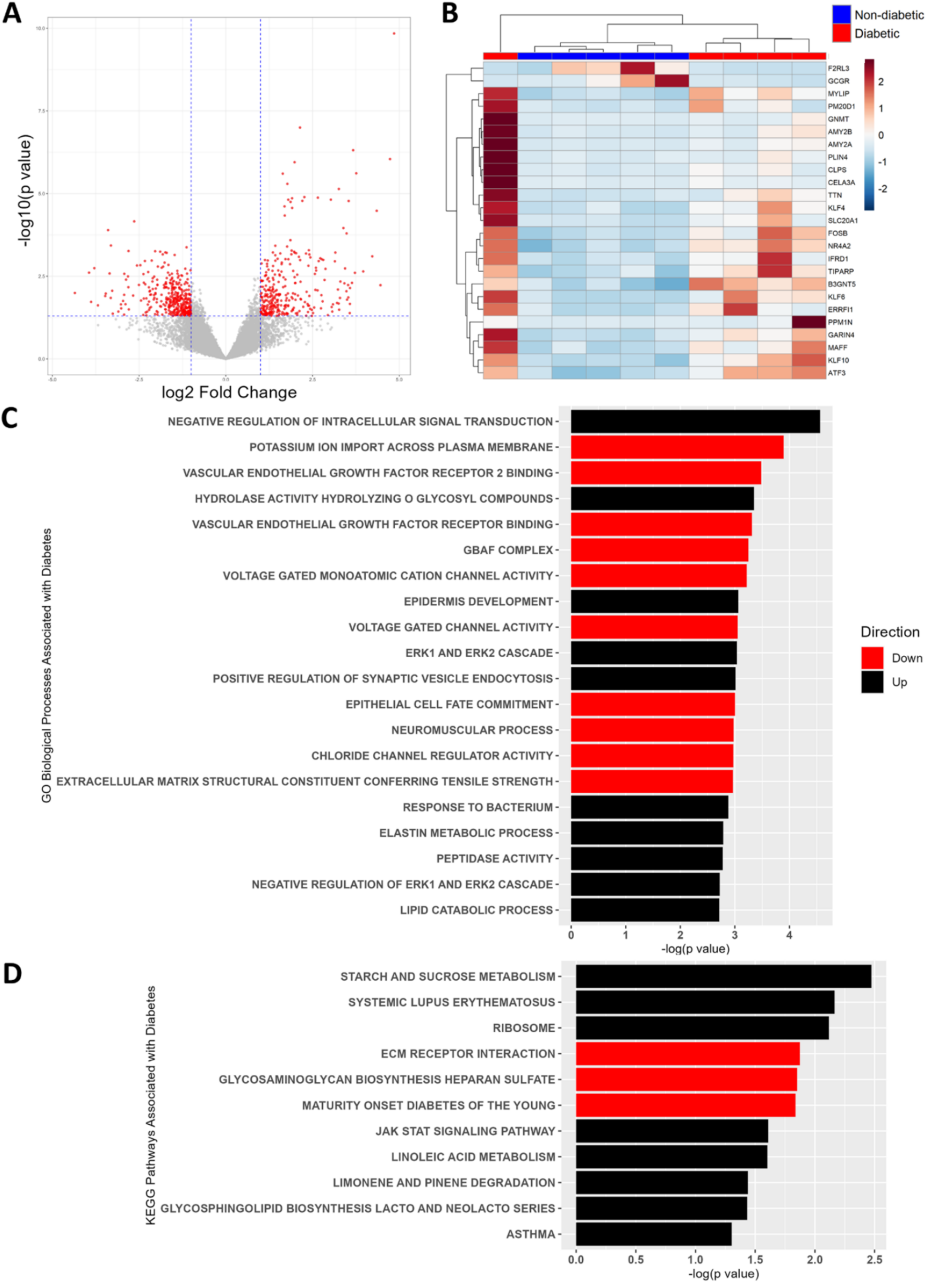
Transcriptomic differences in islets from T2D vs nondiabetic donors. (A) Volcano plot visualizing differential gene expression by −log_10_(*p*-value) from Wald tests vs log_2_(fold change) in T2D compared to nondiabetic islets. Red points represent genes with |fold change| > 2 and *p* < 0.05. (B) Heatmap representation of relative levels of transcripts differentially abundant (FDR *p*-adjusted <0.1) in T2D and nondiabetic islets by the Wald test. Clustering by Ward’s algorithm, expression values scaled by row. (C) Top 10 up- and 10 downregulated Gene Ontology (GO) Biological Processes and (D) Kyoto Encyclopedia of Genes and Genomes (KEGG) Pathways enriched in genes differentially expressed (*p* < 0.05) between T2D and nondiabetic islets, derived from gene set enrichment analysis. Genes were ranked by the product of their log_2_(fold change) in expression between T2D and nondiabetic islets and their log_10_(*p*-value) derived from Wald tests. Red bars indicate Processes/Pathways with enrichment scores (ESs) < 0, indicating enrichment in genes with lower expression in T2D islets. Those with ESs > 0 are depicted in black.

Enrichment analysis of the full transcriptomics data set based on GO and KEGG terms revealed 491 and 11 significantly impacted Biological Processes or Pathways (*p* < 0.05), respectively (Figure 1C,D and Table S2). None were significant after FDR adjustment (adjusted *p* < 0.1). Several of the top Processes and Pathways were related to cell signaling, extracellular matrix, immune activity, ion channel activity, and lipid metabolism, processes frequently implicated in T2D pathology. Another notable hit was the gene cluster related to Maturity-Onset Diabetes of the Young, a group of rare genetic forms of diabetes. Expression of a number of genes in this pathway (*NKX2*-*2*, *NEUROD1*, *GCK*, *PDX1*, *HNF1A*), which are known to regulate β cell development, mature phenotype, and insulin secretion,^53^ was reduced in T2D islets.

Similar physiological processes were among the top results in enrichment analysis after exclusion of the sample, which included acinar pancreatic cells, and one KEGG Pathway, extracellular matrix receptor interaction, was significant after FDR adjustment (adjusted *p* = 0.08) (Table S2).

### Proteomics Largely Highlighted Known Processes Related to T2D β Cell Dysfunction and Displayed Limited Overlap with Gene Expression Data

Proteomic profiles were obtained from three diabetic and three nondiabetic donors. Of 5323 proteins characterized quantitatively, 138 were differentially abundant (*p* < 0.05) between T2D and nondiabetic islets, with 60 more abundant in T2D islets and 78 less abundant (Figure 2A and Table S3). Thirty remained significant after correction for FDR (adjusted *p* < 0.1) (Figure 2B). Top differentially abundant proteins by *p*-values were POLR2G, AFAP1L2, SIGMAR1, NR2C2AP, MRPL23, ENG, UIMC1, and NIP7 (Table S3).

**Figure 2:**
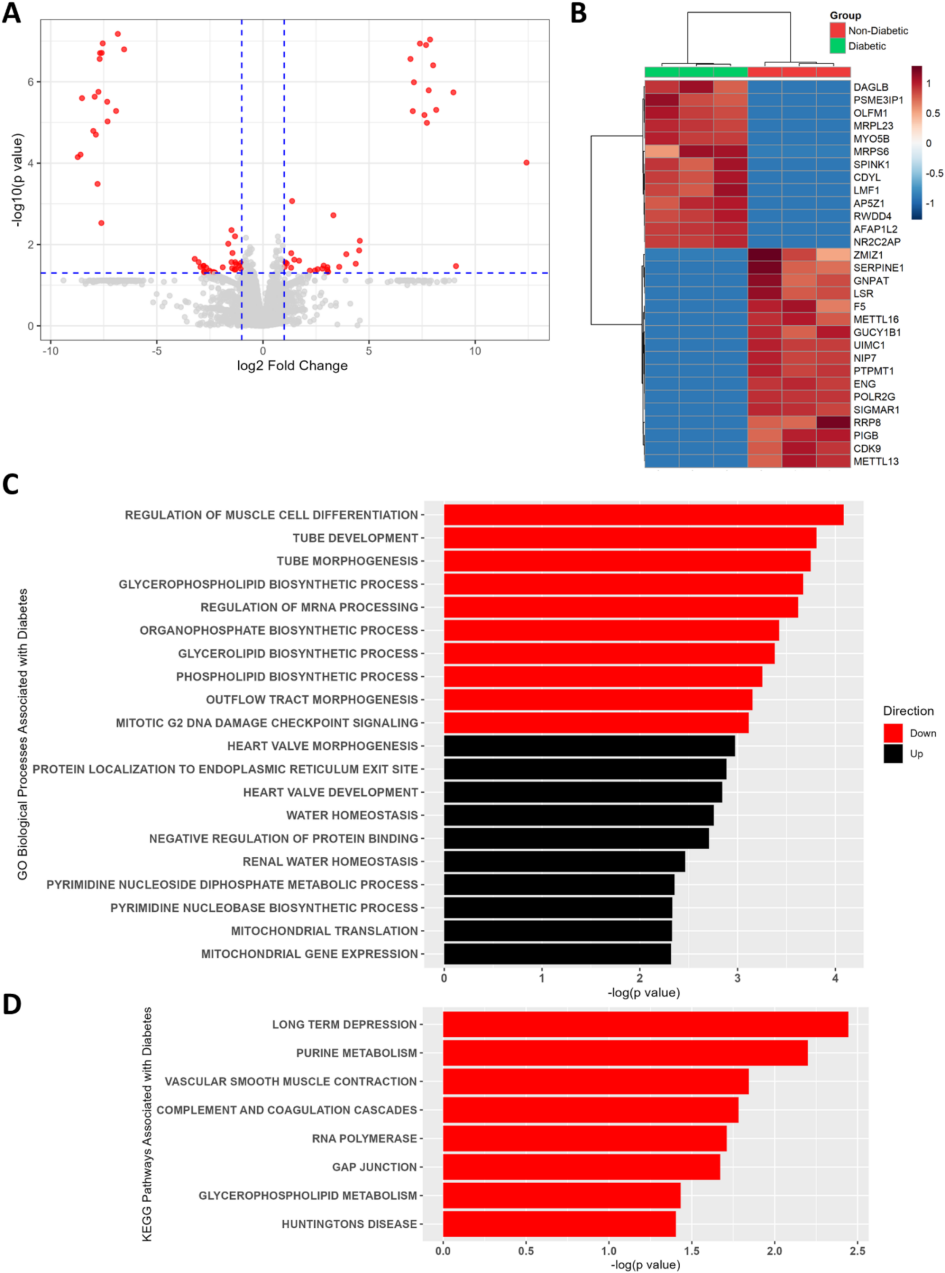
Proteomic differences in islets from T2D vs nondiabetic donors. (A) Volcano plot visualizing protein differential abundance by −log_10_(*p*-value) from limma vs log_2_(fold change) in log_10_-transformed protein heavy/light (H/L) ratios in T2D compared to nondiabetic islets. Red points represent proteins with |fold change| > 2 and *p* < 0.05. (B) Heatmap of relative levels of proteins differentially abundant (limma FDR-adjusted *p* < 0.1) in T2D and nondiabetic islets. Proteins are identified by gene symbol for legibility. Clustering by Ward’s algorithm, log-transformed protein H/L ratios scaled by row. (C) Top 10 up- and 10 downregulated Gene Ontology (GO) Biological Processes and (D) Kyoto Encyclopedia of Genes and Genomes (KEGG) Pathways enriched in proteins differentially abundant (*p* < 0.05) between T2D and nondiabetic islets, derived from gene set enrichment analysis. Proteins were ranked by the product of their log_2_(fold change) in normalized H/L ratios between T2D and nondiabetic islets and their log_10_(*p*-value) derived from limma. Red bars indicate Processes/Pathways with enrichment scores (ESs) < 0, indicating enrichment in proteins with lower abundance in T2D islets. Those with ESs > 0 are depicted in black.

Enrichment analysis yielded no results significant at FDR-adjusted *p* < 0.1; 243 GO Biological Processes and 8 KEGG Pathways were identified at *p* < 0.05. The top Processes and Pathways were largely related to cell proliferation, cell differentiation, transcription, lipid biosynthesis (phospholipids, glycerolipids, and glycerophospholipids), protein trafficking and exocytosis, purine and pyrimidine metabolism, and mitochondrial function (Figure 2C,D and Table S4), most of which relate to known β cell dysfunction in T2D.

Despite the smaller sample size for proteomics, five features were found to differ significantly in abundance (*p* < 0.05) of both transcripts and proteins (Tables S1 and S3). ATF3 and CYP4X1 were both more abundant in T2D; LRRC20 and LRSAM1 were less abundant. ELOC (or TCEB1) was significantly differentially abundant in both data sets; however, its gene expression was increased, while its protein level was reduced in islets from donors with T2D. Of these proteins and transcripts, only *ATF3* was differentially abundant after adjustment for FDR.

Excluding the sample containing some acinar pancreatic cells from the transcriptomic data set did not markedly improve alignment between the transcriptomic and proteomic results. ATF3, LRRC20, and LRSAM1 remained concordant, while CYP4X1 and ELOC were no longer statistically differentially abundant in the transcriptomics data subset. Additionally, MROH1 and CDK9 were less abundant in both proteomic and the smaller transcriptomics data sets, ZC3H11A was more abundant, and AP5Z1 and TMUB1 were differentially abundant by both omics approaches; however, their gene expression was reduced, while their protein level was increased in islets from donors with T2D (Tables S1 and S3).

### Untargeted Metabolomics Suggested That Industrial Chemicals and Pesticides/herbicides Were More Abundant and That Numerous Metabolic Pathways Were Perturbed in T2D Islets

Of 8842 metabolomic features, 542 differed significantly (*p* < 0.05) in intensity between T2D and nondiabetic islets (Figure 3A and Table S5). Thirty features—four of which were annotated by *mummichog*, T3DB, or both—remained significant following adjustment for FDR (adjusted *p* < 0.1) (Figure 3B). Three of the four were less abundant in islets from T2D donors; these were annotated as acetyl-CoA, 4-imidazolone-5-propanoate or imidazole-4-acetaldehyde, and flame-retardant octabromobiphenyl ether (T3DB) or a leukotriene, lipoxin, or prostaglandin H3 (PGH3; *mummichog*). The one feature more abundant in T2D was annotated as flame-retardant octabromobiphenyl (T3DB) or heme O (*mummichog*).

**Figure 3:**
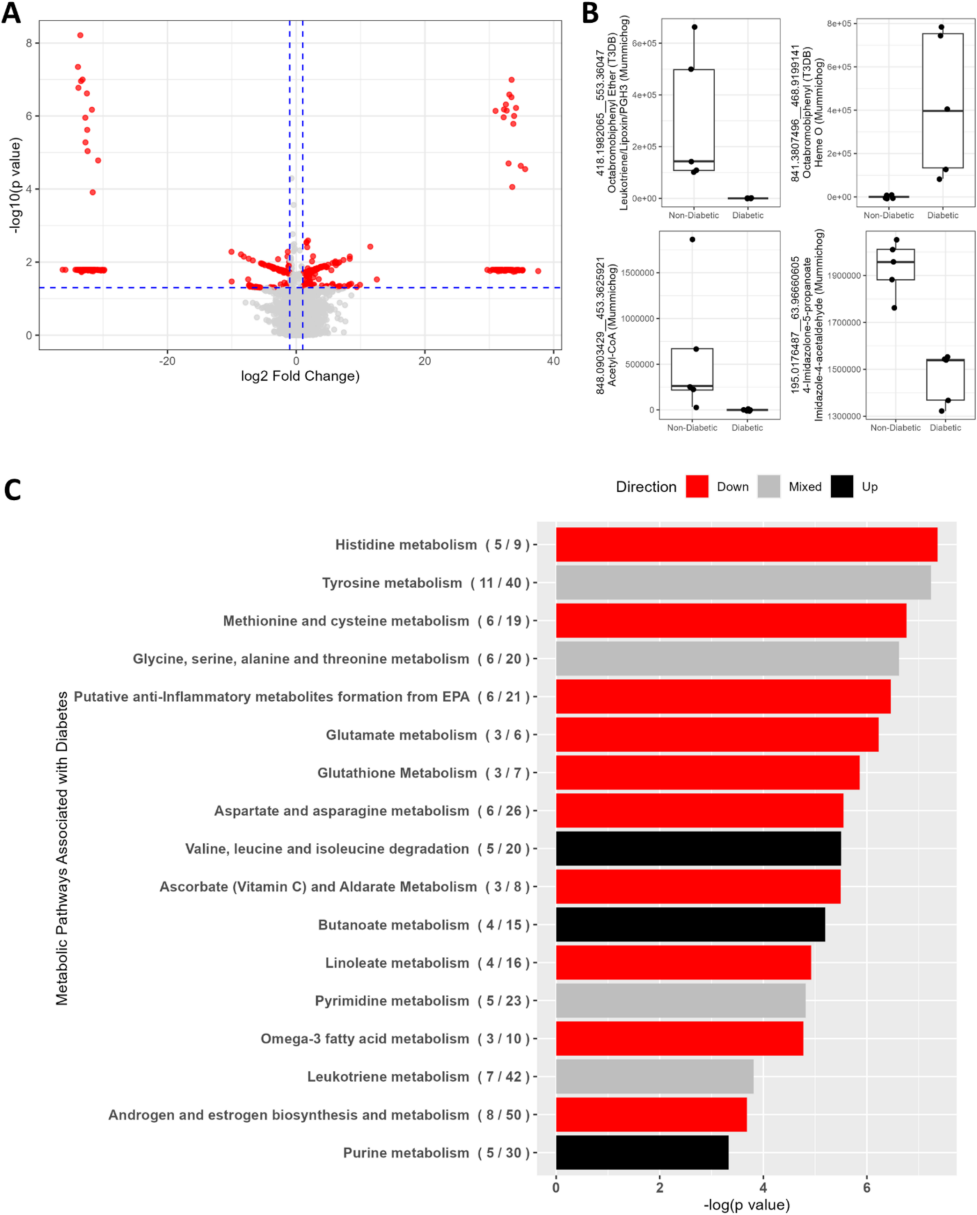
Metabolomic differences in islets from T2D vs nondiabetic donors. (A) Volcano plot visualizing differential metabolomic feature intensity by −log_10_(*p*-value) from *t* tests vs log_2_(fold change) in T2D compared to nondiabetic islets. Red points represent features with |fold change| > 2 and *p* < 0.05. (B) Intensity values of annotated metabolomic features differentially abundant in T2D and nondiabetic islets at FDR-adjusted *p* < 0.1 by *t* tests. Features are identified by the mass:charge ratio (*m*/*z*) and retention time (rt; *m*/*z*_rt) and by annotations from the Toxin and Toxin Target Database (T3DB) and *mummichog*. (C) Metabolic pathways enriched (*p* < 0.05 and at least three differentially abundant metabolites) in metabolites differentially abundant (*t* test *p* < 0.05) between T2D and nondiabetic islets. Pathway *p*-values assigned by *mummichog* account for the number of differentially abundant metabolites assigned to a pathway relative to the total number of metabolites assigned to that pathway [(# differentially abundant metabolites/# total metabolites)]. Red bars and black bars indicate pathways enriched in metabolites less abundant or more abundant, respectively, in T2D islets compared to nondiabetic. Gray bars indicate a mixture of metabolites more and less abundant in T2D islets assigned to that pathway.

When the sample containing acinar pancreatic cells was excluded from the analysis, 49 metabolomic features were statistically differentially abundant (adjusted *p* < 0.1) between T2D and nondiabetic islets. This included the four annotated features identified in analysis of the entire data set along with another feature more abundant in T2D samples annotated as octabromobiphenyl, a feature more abundant in T2D samples annotated as an antipsychotic medication, and a feature less abundant in T2D samples identified as a fungicide (Table S5).

Among the 542 features differentially abundant at *p* < 0.05 in the entire data set, a subset also matched entries in T3DB, several of which also had alternative annotations assigned by *mummichog* (Table S5). Islets from diabetic donors contained higher levels of features annotated as industrial chemicals, adhesives, phthalates, and herbicides. Islets from nondiabetic donors contained higher levels of features annotated as fungicides; a dioxin; or chemicals used in paints, lacquers, cosmetics, and cleansers. These patterns remained consistent when the sample containing acinar pancreatic cells was excluded from the analysis.

Metabolic pathways represented by the features differentially abundant at *p* < 0.05 (Figure 3C and Table S6) were largely related to amino acid metabolism and lipid metabolism. The majority of these pathways included features that were significantly (*p* < 0.05) less abundant or undetected in diabetic islets. Additional pathways included pyrimidine and purine metabolism, ascorbate (vitamin C) and aldarate metabolism, and androgen and estrogen biosynthesis and metabolism. The last three of these pathways were no longer identified when the sample containing acinar pancreatic cells was excluded, but other pathway analysis results were largely unchanged.

### Differential Network Analysis

#### Connectivity of Omics Features Differed between T2D and Nondiabetic Networks

Two networks were constructed, one from T2D islet data and the other from nondiabetic islet data, integrating transcriptomics, proteomics, and metabolomics and showing the most closely associated features by abundance patterns across samples (Figure 4A). The nondiabetic network included 2652 metabolites (our selected maximum), 3097 transcripts, and 1596 proteins (our selected maximum), with five clusters or communities identified. The diabetic network included 2652 metabolites (the maximum), 3519 transcripts, and 1596 proteins (the maximum), comprising six communities. More than half the proteins were represented in both networks, along with over a third of the metabolites and roughly a third of the transcripts.

**Figure 4:**
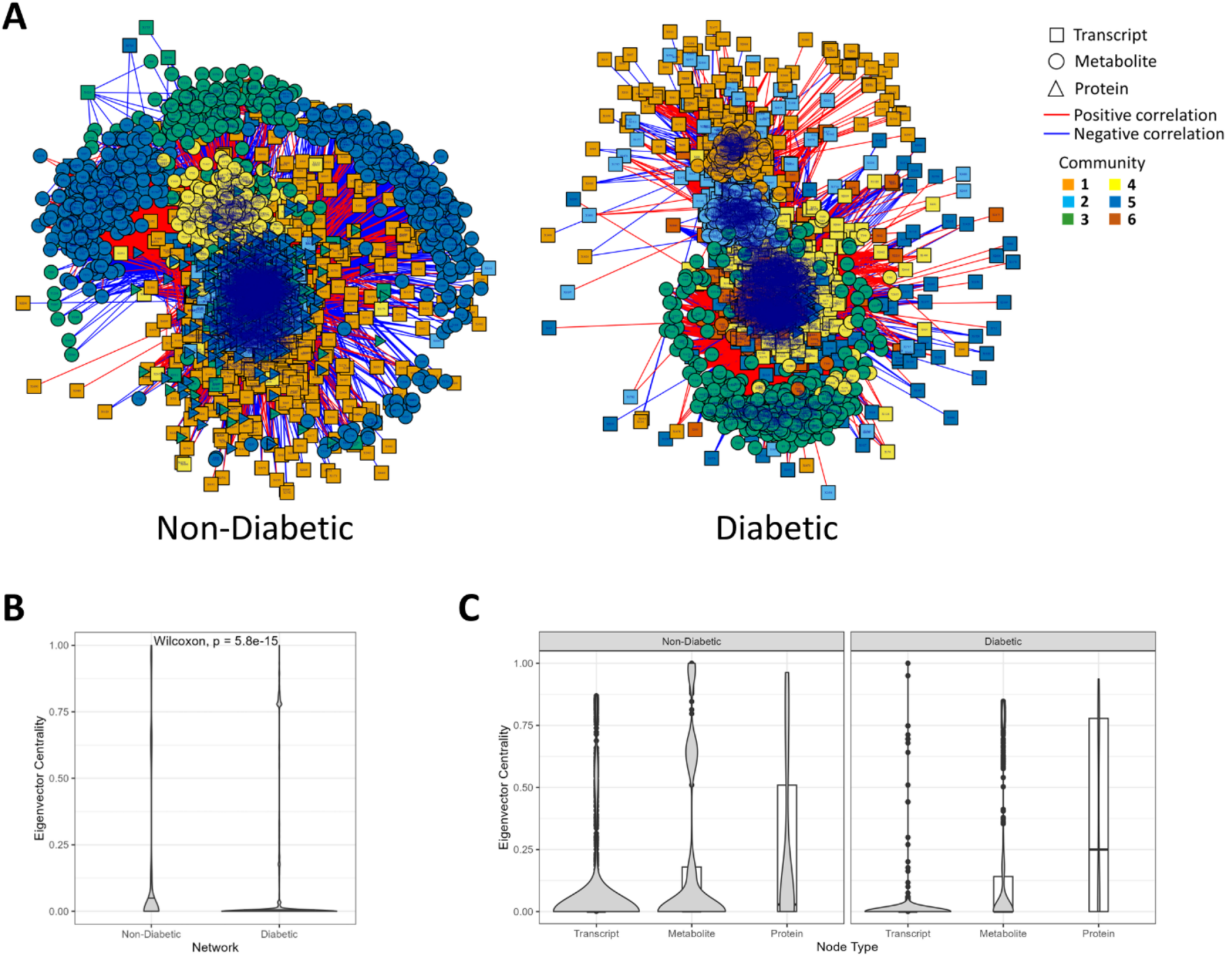
Differential network analysis of integrated transcriptomics, metabolomics, and proteomics from islets of T2D vs nondiabetic donors. (A) Networks of associated metabolites (circles), transcripts (squares), and proteins (triangles) in islets from nondiabetic donors (left) and donors with T2D (right). Node colors represent communities of the most closely related omics features, and edge colors represent the direction of correlation between nodes. (B) Violin plot of eigenvector centrality scores for features included in the nondiabetic and T2D networks, with comparison by the Wilcoxon rank sum test. Features with greater connectivity within the network had higher eigenvector centrality scores. (C) Violin with overlaid boxplot of eigenvector centrality scores of transcripts, metabolites, and proteins within T2D and nondiabetic networks.

Features in the nondiabetic network were more evenly distributed, with higher median eigenvector centrality, than those in the diabetic network, which contained many nodes with minimal connectivity to other nodes along with clusters of tightly correlated features (Figure 4B). This suggests that the network of molecular interactions in islets is disrupted in T2D and that groups of related molecules representing a few biochemical or cellular processes have a strong influence on the abundance of other molecules.

Differential network analysis excluding the sample containing some acinar pancreatic cells produced similarly structured networks (Figure S1A), but the difference in the eigenvector centrality between the networks was no longer statistically significant (Figure S1B).

#### Transcripts Were Less Influential Than Proteins or Metabolomic Features in Both Networks

In both T2D and nondiabetic networks, most transcripts had low centrality scores relative to proteins or metabolites (Figure 4C). Of over 3000 transcripts in each network, just 341 differed substantially (>0.1) in centrality between the two networks, indicating a marked divergence in their connectivity between the networks. GSEA yielded no Processes or Pathways significant at FDR-adjusted *p* < 0.1 for the set. Although 319 GO Biological Processes and 4 KEGG Pathways were identified at *p* < 0.05, most of these were identified on the basis of just one or two transcripts with |delta centrality| > 0.1. Several transcripts linked to the top Biological Processes/Pathways (Table S7) were associated with cytoskeletal function; cell proliferation; β cell differentiation and function including insulin secretion; and immune responses including immune cell migration, phagocytosis, and antigen receptor signaling. Additional transcripts were associated with fatty acid transport, cholesterol metabolism, protein trafficking, apoptosis, RNA processing, and mitochondrial function.

Exclusion of the sample containing some acinar pancreatic cells from the integrated analysis did not substantially impact the contributions of transcriptomic features to the networks. Just 106 transcripts differed in centrality (>0.1) between the two networks, and enrichment for no Biological Processes/Pathways was identified among them at FDR-adjusted *p* < 0.1 (Figure S1C and Table S7).

#### Proteins That Differed in Connectivity in T2D vs Nondiabetic Networks Were Primarily Related to Immune Responses

Proteins had the highest median centrality scores in the network analysis (Figure 4C), and 1272 presented with |delta centrality| > 0.1 between the T2D and nondiabetic networks. These were related to 8 GO Biological Processes and 13 KEGG Pathways (FDR-adjusted *p* < 0.1) (Figure 5A). Proteins associated with these Processes/Pathways (Table S8) included several functioning in cell proliferation and differentiation and in energy metabolism including lipid metabolism, glucose metabolism, and insulin secretion, but the majority were related to immune responses. These included proteins expressed on T cells, B cells, and myeloid cells and associated with immune cell adhesion and infiltration, oxidative stress, phagocytosis, antigen presentation, and antiviral responses.

**Figure 5:**
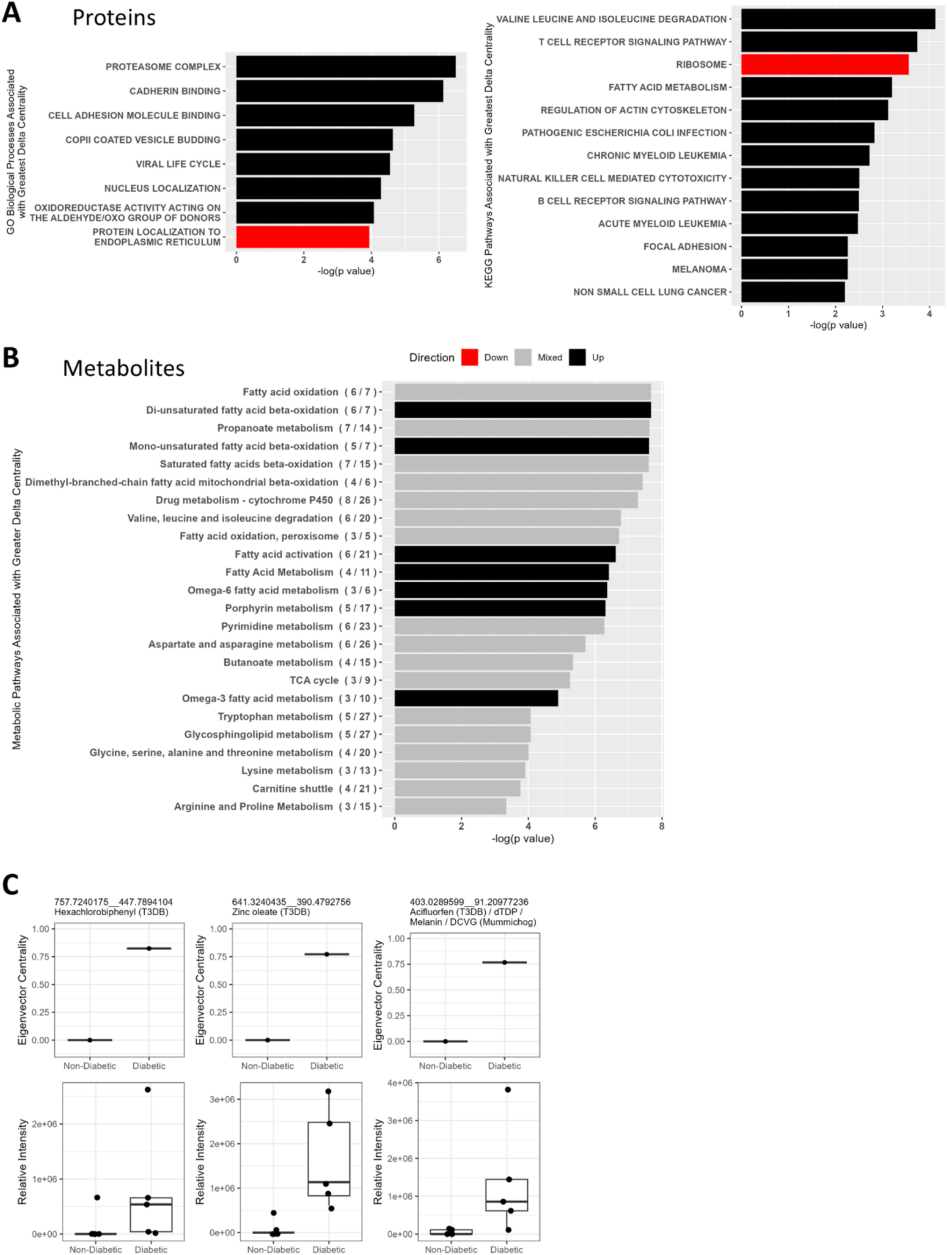
Processes, pathways, and features with a substantial difference in eigenvector centrality between T2D and nondiabetic integrated omics networks. (A) Gene Ontology (GO) Biological Processes and Kyoto Encyclopedia of Genes and Genomes (KEGG) Pathways enriched (FDR-adjusted *p* < 0.1) in proteins with a difference in eigenvector centrality (delta centrality) >0.1 or <−0.1 between T2D and nondiabetic networks, derived from gene set enrichment analysis. Proteins were ranked by delta centrality. Red bars indicate Processes/Pathways with enrichment scores (ES) < 0, indicating enrichment in proteins with lower centrality in the T2D network. Those with ES > 0 are depicted in black. (B) Metabolic pathways enriched (*p* < 0.05 and at least three differentially abundant metabolites) in metabolites with |delta centrality| > 0.1 between T2D and nondiabetic networks, indicating pathways differentially prominent within the networks. Pathway *p*-values assigned by *mummichog* account for the number of differentially abundant metabolites assigned to a pathway relative to the total number of metabolites assigned to that pathway [(# differentially abundant metabolites/# total metabolites)]. Black bars indicate pathways enriched in metabolites with higher centrality in the T2D network. Gray bars indicate a mixture of metabolites with centrality >0.1 and centrality <−0.1 in the T2D network assigned to that pathway. (C) eigenvector centrality and intensity values of putative hazardous chemicals which were more central in the T2D network (delta centrality >0.1) and also more abundant in islets from diabetic vs nondiabetic donors (*t* test *p* < 0.05); features are identified by mass:charge (*m*/*z*) and retention time (rt; *m*/*z*_rt) and by annotations from the Toxin and Toxin Target Database (T3DB) and *mummichog*.

When the sample containing some acinar pancreatic cells was excluded from the network analysis, the 1177 proteins with |delta centrality| > 0.1 were enriched (FDR-adjusted *p* < 0.1) in markers connected to 20 GO Biological Processes and 11 KEGG Pathways. These included most of the originally identified Processes/Pathways and additional ones related to translation, signal transduction, and protein secretion (Figure S1D and Table S8).

#### Metabolomic Features Linked to Fatty Acid and Porphyrin Metabolism Were More Influential in the T2D Network

Between the two networks, 1500 metabolomic features had |delta centrality| > 0.1. The metabolic pathways represented by these metabolites are related to lipid metabolism, pyrimidine metabolism, tricarboxylic acid (TCA) cycle, drug metabolism, and porphyrin metabolism (Figure 5B and Table S9). Fatty acid activation, fatty acid metabolism, mono- and diunsaturated fatty acid β-oxidation, omega-3 and omega-6 fatty acid metabolism, and porphyrin metabolism pathways were generally more influential in the diabetic network, with at least 80% of their associated metabolites with |delta centrality| > 0.1 having higher centrality in the diabetic network.

When the sample containing some acinar pancreatic cells was excluded, 948 metabolomic features had |delta centrality| > 0.1, representing 18 biochemical pathways, including the majority of those identified originally (Figure S1E and Table S9). Pathways no longer selected in this analysis of the smaller data set included several related to amino acid metabolism, carnitine shuttle, and butanoate metabolism. Pathways newly identified in this analysis were purine metabolism, leukotriene metabolism, amino sugar metabolism, and bile acid biosynthesis (Table S9).

#### Select Putative Hazardous Chemicals Were Differentially Positioned in T2D and Nondiabetic Networks

In addition, regardless of whether or not the sample containing acinar pancreatic cells was included in the analysis, three metabolomic features that were more central in the diabetic network (delta centrality >0.1) and also more abundant in diabetic islets (*p* < 0.05) matched entries in T3DB (Figure 5C), suggesting that they could be hazardous chemicals potentially contributing to T2D islet pathology. These features were annotated as industrial additives, hexachlorobiphenyl and zinc oleate, and the herbicide, acifluorfen. The molecule annotated as acifluorfen by T3DB was alternatively annotated by *mummichog* as deoxythymidine diphosphate (dTDP), melanin, or S-(1,2-dichlorovinyl)­glutathione (DCVG).

To obtain information about potential interactions of these molecules in T2D islets, we examined the nodes with which they were connected in the diabetic network. All three of these putative hazardous chemicals had linkages similar to those of a set of proteins. GSEA indicated that these linkages represented inverse associations between levels of the chemical and protein translation and associations with mitochondrial function, regulation of gene expression, and fatty acid metabolism (Table S10).

## Discussion

Our understanding of mechanisms driving T2D pathology within the pancreatic islet β cells remains incomplete but could be further enhanced by multiomics profiling. To our knowledge, this is the first study to simultaneously evaluate transcriptomic, proteomic, and metabolomic profiles of islets from donors with T2D and compare these with islets from nondiabetic donors utilizing multiomics integration and network analysis.

Supporting the validity of our analysis, even in a small number of samples, many of our findings have clear physiological connections to known T2D islet pathology, such as changes in β cell mass, dedifferentiation, insulin secretion, and oxidative stress. Additionally, molecules and pathways related to lipid metabolism were identified in analyses of all three data sets and also represented among the features that differed most in connectivity in the T2D and nondiabetic networks, suggesting that they are both differentially active and differentially influential in T2D islets. Alterations in lipid and fatty acid metabolism have long been recognized in T2D.^54^ Our metabolomic and network analyses also indicated the perturbation, and likely suppressed activity, of numerous pathways related to amino acid metabolism. It is known that exposure to certain amino acids can stimulate or enhance insulin secretion from pancreatic β cells.^55^

On the other hand, high levels of branched-chain amino acids (BCAAs), including valine, leucine, and isoleucine, in circulation are associated with obesity and metabolic disorders including T2D.^56^ Metabolism of these BCAAs was predicted to be elevated in T2D islets in our study, and BCAA metabolism was differentially influential in T2D vs nondiabetic islets in our network analysis, although not when the sample containing some acinar pancreatic cells was excluded. BCAAs are known to regulate insulin sensitivity and lipid metabolism in skeletal muscle, but their activity and that of other amino acids in pancreatic islets require further investigation.^56^

We identified other processes with connections to T2D pathophysiology that have not been well-characterized in pancreatic islets, including purine and pyrimidine, porphyrin, and histidine metabolism and immune activity. Purine and pyrimidine metabolism differed in our proteomics and metabolomic profiling of T2D islets, and pyrimidine metabolism was also predicted to be differentially influential in T2D islets in the network analysis. When the sample containing some acinar pancreatic cells was excluded from analysis, purine metabolism was also identified as being differentially influential. Alterations in purine metabolism, in particular, have been examined in relation to T2D,^57^ but the impact in islets has not been established. These nucleotide-related pathways are closely linked with energy homeostasis through their utilization of, contributions to synthesis of, and response to adenosine triphosphate (ATP),^58^ which regulates β cell function and specifically insulin secretion.^57^ The molecule acetyl-CoA acts as a hub in the TCA cycle for ATP synthesis, but it also intersects with numerous other pathways including synthesis and catabolism of fatty acids and certain amino acids, serving to coordinate their activity with cellular metabolism.^59^ We found that a metabolomic feature annotated as acetyl-CoA was markedly less abundant in the T2D islets. Its depletion in β cells in T2D would align with a fundamental disruption of the energy metabolism.

Another top differentially abundant metabolite that was more abundant in T2D islets was annotated as heme O. Furthermore, metabolites related to porphyrin metabolism were more central in T2D than the nondiabetic network. Porphyrins are precursors of heme, and heme is predominantly synthesized in mitochondria.^60^ As mitochondrial activity is disrupted in β cells in T2D, synthesis of heme and associated iron trafficking in these cells may also be perturbed, promoting oxidative stress through heme’s actions as a redox cofactor and potentially ferroptosis.^60^ Our results suggest that these processes could be influential in T2D islets.

An intriguing finding was the reduction in a metabolite annotated as 4-imidazolone-5-propanoate or imidazole-4-acetaldehyde in T2D islets. These molecules are derived from histidine. Recently, a closely related molecule, imidazole propionate, was found to be elevated in circulation in individuals with T2D^61,62^ and to impair glucose tolerance and insulin signaling in the liver.^61^ Increased levels of this molecule were attributed to altered microbial metabolism of histidine.^61,62^ While imidazole propionate itself was not detected in our data sets, this study provides the first indication that altered histidine metabolism, the top pathway identified in our metabolomic analysis, may be impacting β cell function directly rather than just through systemic effects or effects on the liver.

Immune dysregulation is an established component of T2D, but the immune cell types involved and the nature of their interactions with β cells remain unclear.^63–65^ We found that immune-related proteins and transcripts more central in the T2D network represented a range of immune cells and functions linked to both innate and adaptive immunity. Importantly, immune activity did not feature prominently in the pathways represented by differentially abundant proteins in the individual proteomics analysis; rather, we found differences in the connectivity of immune-related markers within T2D vs nondiabetic omics feature networks. This suggests that even in the absence of a clear inflammatory signature detected in islets, immune responses may be key drivers of the T2D islet cell phenotype and worthy of further investigation as therapeutic targets. This finding demonstrates the value of our integrative multiomics approach in yielding mechanistic insights into islet pathology in T2D.

Recently, a multiomics study of pancreatic islets by Kolic et al. found relatively little overlap between transcriptomics and proteomics data.^17^ Similarly, we identified just a few features that differed significantly in islets from diabetic donors at the transcript and protein level. In further corroboration of Kolic et al., our network analysis showed that transcripts were not as highly correlated with other features as were proteins or metabolites. Relatively few transcripts exhibited large differences in centrality between T2D vs nondiabetic networks, and no significant enrichment of these transcripts in GO Biological Processes or KEGG Pathways was found after FDR correction. This suggests that alterations in gene expression are not the primary drivers of T2D-specific changes in the islet’s molecular environment.

A final notable finding from this study was the indication that environmental chemical exposure may be influencing molecular interactions in islets in T2D. Three metabolomic features found to be more abundant in T2D islets (undetected in most nondiabetic samples) and more centrally positioned in the T2D network were annotated as industrial chemicals, hexachlorobiphenyl and zinc oleate, and as the herbicide, acifluorfen. Furthermore, some of the most differentially abundant metabolites were annotated as octabromobiphenyl or octabromobiphenyl ether, which are components of flame retardants that can act as endocrine disrupters. Recent studies have linked other brominated flame retardants to T2D risk, making the potential detection of these molecules in pancreatic islets intriguing and concerning.^66,67^

Both hexachlorobiphenyl and acifluorfen are organochlorine compounds, and higher levels of organochlorine pollutants, particularly hexachlorobiphenyls, in human serum have been linked to T2D.^68–70^ A rodent study demonstrated that these compounds could alter insulin activity and perturb lipid metabolism, mitochondrial function, and immune activity in the liver.^71^ Furthermore, the activity of hexachlorobiphenyl and acifluorfen also influences porphyrin metabolism,^72^ and proposed mechanisms mediating connections between organochlorine compounds and T2D are oxidative stress and mitochondrial dysfunction.^70^ Indeed, when we examined the nodes with the closest connections to these metabolites in the diabetic network, we found that they were proteins involved in mitochondrial function and lipid metabolism, many with close biochemical relationships with acetyl-CoA. We found inverse associations between levels of these potentially hazardous chemicals and ribosomal proteins involved in translation, supporting the idea that the documented systemic or hepatic effects of these chemicals may also be occurring in pancreatic islets themselves in T2D. Exposure to these and other endocrine-disrupting compounds may represent an underappreciated risk factor for T2D.

This pilot study has clear limitations. A small sample size limits its generalizability and the potential to identify true positive signals in high-dimensional data and precludes the development of multivariate models. Additionally, the diabetic and nondiabetic donors were also not matched for demographic or basic clinical characteristics such as sex, age, or BMI, which may have obscured some disease-specific differences. Finally, with untargeted omics, identification of the detected molecules, particularly metabolites, is not absolute and is not always possible without additional biochemical analyses. This study tested the potential value of this multiomics approach, and it identified candidate metabolites, transcripts, and proteins distinguishing T2D islets; however, these candidates require confirmation in future investigations.

## Conclusions

This multiomics analysis corroborated many of the mechanisms reportedly involved in islet cell dysfunction in T2D and examined their relative influence in a molecular network, identifying central processes driving islet dysfunction in T2D. We also identified other pathways worthy of further study in islets and the effects of specific classes of potentially hazardous chemicals. Multiomics can maximize the information obtained from rarely accessible pancreatic islets and has the potential to provide novel insights into T2D pathology.

## Supplementary Material



## Notes

Tables detailing all differentially abundant transcripts, proteins, and metabolites identified in these analyses along with associated statistics, all related processes and pathways identified in enrichment analyses, and a figure reflecting the results of the integrated multiomics analysis excluding one islet sample that appeared to contain an over-representation of acinar pancreatic cells in addition to β cells (XLSX)

## ○

Department of Radiation Oncology, City of Hope National Medical Center, 1500 E Duarte Road, Duarte, California 91010, United States

## ◆

Department of Biomedicine, Aarhus University, Nordre Ringgade 1, 8000 Aarhus Centrum, Denmark.

## ¶

Institute of Translational Proteomics, Philipps-Universität Marburg, Karl-von-Frisch-Straße 2, 35043 Marburg, Germany.

## ⋈

Professor Emeritus.
